# Generalization of threats attributed to large carnivores in areas of high human–wildlife conflict

**DOI:** 10.1111/cobi.13974

**Published:** 2022-08-04

**Authors:** Kumar Ashish, Tharmalingam Ramesh, Riddhika Kalle, Rathinasamy Arumugam

**Affiliations:** ^1^ Division of Conservation Ecology Sálim Ali Centre for Ornithology and Natural History Coimbatore India; ^2^ Centre for Functional Biodiversity, School of Life Sciences University of KwaZulu‐Natal, Private Bag X01 South Africa; ^3^ Division of Environmental Impact Assessment Sálim Ali Centre for Ornithology and Natural History Coimbatore India; ^4^ Chinmaya Vidyalaya PACR Matriculation Higher Secondary School Rajapalayam India

**Keywords:** false memory, generalization, human–carnivore conflict, hyena, livestock predation, random forest, tolerance, bosque aleatorio, conflicto humano‐carnívoros, depredación de ganado, generalización, hiena, memoria falsa, tolerancia, 错误记忆, 泛化, 人类与食肉动物的冲突, 家畜捕食, 鬣狗, 容忍, 随机森林

## Abstract

Fear‐induced generalization of threats to noninimical stimuli is a behavioral tendency of humans to minimize exposure to potential threats. In human–carnivore conflict zones, people often generalize their fear of predation by obligate carnivores to nonobligate carnivores despite differences in species’ predation rates. We investigated the effect of a perceived threat of large obligate carnivores to livestock on tolerance and perception of striped hyena (*Hyaena hyaena*) in an area of high human–carnivore conflict. We surveyed 197 households through asemistructured questionnaire to determine people's perception and tolerance of striped hyenas in Sathyamangalam and Mudumalai Tiger Reserves after identification of the current distribution range of hyena determined through camera trap and sign surveys. Through the random forest algorithm, we modeled the level of tolerance of striped hyena as a function of loss of livestock to predation and from disease, the perceived threat of predation by hyena, and other socioeconomic attributes. Animal husbandry was a major source of income but was severely affected by livestock loss due to predation and disease. Sixty‐nine percent of people were uncertain about predatory behavior of hyena; out of that, 23% reported a negative conservation attitude. Only 6 instances of hyena depredation on livestock and 2 on dogs were reported. Our model confirmed that economic instability associated with increased loss to predation and disease, livestock dependency, and a decrease in family annual income negatively affected people's tolerance of hyena. Perceptual uncertainty related to predatory behavior of hyena also negatively affected people's tolerance. In our study area, economic instability and perceptual uncertainty led to generalization of fear of large carnivores to a nonobligate predator. Such generalization may affect the attitude of people toward many other species. Understanding the role of economic instability and perceptual uncertainty should facilitate conservation of species, such as the hyena, that are vulnerable to false generalization.

## Abstract (es)

Generalización de las Amenazas Atribuidas a los Grandes Carnívoros en las Áreas con un Elevado Conflicto Humano‐Fauna

**Resumen:**

La generalización de las amenazas inducidas por el miedo a los estímulos no desfavorables es una tendencia del comportamiento humano para minimizar la exposición ante las amenazas potenciales. En las zonas de conflicto humano‐carnívoros, las personas transfieren con frecuencia su miedo a los carnívoros obligados hacia los no obligados a pesar de las diferencias en las tasas de depredación de las espcies. Investigamos el efecto de la amenaza percibida que representan los grandes carnívoros obligados para el ganado sobre la tolerancia a y la percepción de la hiena rayada (*Hyaena hyaena*) en un área con un conflicto elevado entre humanos y carnívoros. Encuestamos a 197 hogares con un cuestionario semiestructurado para determinar la percepción y tolerancia que tienen las personas hacia la hiena rayada en las reservas de tigres de Satayamangalam y Mudumalai después de identificar la distribución actual de la hiena mediante cámaras trampa y censos de indicios. Modelamos el nivel de tolerancia a la hiena rayada con el algoritmo de bosque aleatorio como función de la pérdida de ganado por depredación o enfermedad, la amenaza percibida de la hiena como depredador y otros atributos socioeconómicos. La crianza de animales fue una gran fuente de ingresos aunque con severas afectaciones por la pérdida de ganado por depredación o enfermedad. El 69% de las personas tenían incertidumbre acerca del comportamiento de depredación de la hiena; de ese porcentaje, el 23% reportó una actitud negativa de conservación. En cuanto a las hienas, sólo se reportaron seis instancias de depredación de ganado y dos de perros. Nuestro modelo confirmó que la inestabilidad económica asociada con el incremento en las pérdidas por depredación y enfermedad, la dependencia por el ganado y una reducción en el ingreso anual familiar afectó negativamente la tolerancia que tienen las personas hacia las hienas. La incertidumbre percibida relacionada con el comportamiento de depredación de las hienas también afectó negativamente la tolerancia de las personas. La inestabilidad económica y la incertidumbre percibida derivaron en una transferencia del miedo a los grandes carnívoros hacia los depredadores no obligados en nuestra área de estudio. Dicha transferencia puede afectar la actitud de las personas hacia muchas otras especies. El conocimiento sobre el papel que tiene la inestabilidad económica y la incertidumbre percibida debería facilitar la conservación de especies que son vulnerables a las falsas generalizaciones, como es el caso de la hiena.

## 【摘要】

人类在恐惧的诱发下, 具有泛化非有害刺激造成的威胁的行为倾向, 即要尽量减少暴露在潜在威胁中的机会。在人类与食肉动物冲突的地区, 尽管每个物种的捕食率不同, 但人们经常将对专性食肉动物捕食的恐惧泛化到非专性食肉动物之上。本研究调查了在一个人类与食肉动物高度冲突的地区, 人们对于大型专性食肉动物捕食家畜的感知威胁导致的对条纹鬣狗(*Hyaena hyaena*)容忍度和认知的影响。我们通过半结构化问卷调查了197个家庭, 以确定人们对印度Satayamangalam和Mudumalai老虎保护区中条纹鬣狗的认知和容忍度, 此前我们已通过红外相机和标志调查确定了鬣狗当前的分布范围。我们通过随机森林算法, 将对条纹鬣狗的容忍度作为家畜因捕食和疾病造成的损失、感知到的鬣狗捕食威胁, 以及其他社会经济属性的函数进行了建模。畜牧业是这些家庭主要的收入来源, 但由于捕食和疾病造成的家畜损失严重影响了收入。我们发现, 69%的受访者不能确定鬣狗的捕食行为;其中23%的人报告了消极的保护态度。然而, 受访者只报告了6起鬣狗攻击家畜和2起攻击狗的事件。我们的模型证实, 与捕食和疾病损失增加有关的经济不稳定性、对家畜的依赖, 以及家庭年收入的减少, 对于人们是否容忍鬣狗产生了负面影响。与鬣狗捕食行为有关的感知不确定性也对容忍度存在负面影响。在我们的研究地区, 经济不稳定性和感知不确定性导致人们对大型食肉动物的恐惧被泛化到非专性捕食者之中。这种泛化可能影响人们对许多其他物种的态度。理解经济不稳定性和感知不确定性的作用, 有助于保护那些容易被错误泛化的物种(如鬣狗)。

## INTRODUCTION

Globally, carnivore populations inhabit areas proximate to humans, and recurring inimical human–carnivore interactions in such areas spur human–carnivore conflict (HCC) that can lead to, for example, retaliatory killing (Karanth & Madhusudan, 2002). The improvished economy of people susceptible to high HCC, makes livestock losses due to predation, unaffordable and people intolerant of large carnivores and increases negative conservation attitudes toward predators (Graham et al., 2005; Gusset et al., 2009). Retaliation is a manifestation of intolerance that has led to population declines of many species, such as spotted hyena (*Crocuta Crocuta*), tiger (*Panthera tigris*), lion (*Panthera leo*), condor (*Vultur gryphus*), wild dog (*Lycaon pictus*), cheetah (*Acinonyx jubatus*), wolf (*Canis lupus*), and cougar (*Puma concolor*) (Cailly‐Arnulphi et al., 2017; Gusset et al., 2009; Marker et al., 2003). Moreover, this negative conservation attitude can lead to a social repugnance to established conservation policies and can adversely affect wildlife conservation over the long term. Political outrage against wildlife is a clear example of collective and protracted impact of social repugnance in wildlife conservation. In Kenya from 2003 to 2011, 161 lions were killed; 50% of the kills were retaliatory and the rest were due to political outrage (Hazzah et al., 2014). Efforts such as The Great Sparrow Campaign, which has led to the extirpation of sparrows from China, and the extirpation of wolves and cougar from Mexico and Washington state (U.S.A.) (Chapron et al., 2014), exemplify the effect such outrage may have. If social repugnance does not prompt impromptu and direct response, it may embolden outsiders to poach problematic species (Liu et al., 2011). A negative attitude towards conservation that leads to retaliation and social repugnance can have huge negative effects on conservation.

Human tolerance is a function of perception that is determined by experienced loss. Such tolerance varies significantly across demographic characteristics, such as gender, ethnicity (culture and religion), and age (Ballejo et al., 2020; Bhatia et al., 2017).

In conservation science, the term *perception* has relevance as a positive or negative feeling toward wildlife (Cailly‐Arnulphi et al., 2017). Studies have been conducted to understand the mechanism of negative perception toward carnivores (Bhatia et al., 2017; Marker et al., 2003; Mogomotsi et al., 2020; Nawaz et al., 2016). Most of these have explored only the direct impact of socioeconomic factors on perceptions towards carnivores. Such approaches generally fail to provide cogent justifications for negative attitudes and persecutions of carnivores that cause almost negligible economic loss through livestock predation.

All co‐occurring carnivores do not affect people's livelihoods equally (Trajçe et al., 2019). Nonetheless, unsustainable economic loss elicited by a group of species or 1 species can result in people overgeneralizing their predation‐induced fear to other species not associated with large economic losses (Farhadinia et al., 2017). Although higher antagonism towards more problematic species is evident, an increase in intolerance of co‐occurring species has been recorded (Gusset et al., 2009). This underpins the prevalence of generalization of a perceived threat to an entire functional guild. Such overgeneralization of threats contradicts the actual damage caused (Ballejo et al., 2020) and is a threat to the co‐occurring carnivores (Marker et al., 2003). The overgeneralization of fear of predation may occur where there are high levels of economic stress and human–wildlife conflict.

The generalization of threats posed by different species can be understood through the theory of generalization proposed by Shepard (1987). According to which, the distance between a pair of items in psychological space determines whether they will be perceived as similar. Based on this theory, we designed a study to assess the generalization of fear and intolerance of obligate predators (e.g., tiger, leopard [*Panthera pardus*] and dhole [*Cuon alpinus*]) to the striped hyena (*Hyaena hyaena*). In our study area, the leopard, which is similar in body size and weight to hyenas, causes large livestock losses, which may preclude discriminative cognition under high economic stress. Apart from predation, livestock disease also causes large losses and often exceeds the loss from predation in most HCC areas (Harihar et al., 2014). Such bilateral losses may curtail pastoralist sustainability and exacerbate intolerance of carnivores. Therefore, we also assessed the impact of livestock disease on people's tolerance of hyena.

Striped hyena is predominantly a solitary scavenger (Wagner et al., 2008). Its global geographic range includes the Middle East, Caucasus region, Central Asia, and the Indian subcontinent; their southern and western limits are in Africa (Mills & Hofer, 1998). The species has declined sharply throughout its range due to hunting for meat or traditional belief, deliberate poisoning, competition from coexisting carnivores, and reduction in livestock carcasses owing to changes in agropastoral practices (Alam, 2011; Kruuk, 1976; Selvaraj et al., 2018; Wagner, 2006). These threats have led to its extirpation in many places (Hofer & Mills, 1998; Kruuk, 1976), and the species is critically endangered in some areas (Kasparek et al., 2004). The striped hyena population in the Western and Eastern Ghats part of Tamil Nadu (WEGPTN) (Arumugam, 2012) has declined drastically, and presently a small breeding population is confined in the Moyar valley of the Mudumalai–Sathyamangalam landscape, which has high HCC (Ramesh et al., 2020). Information on the reasons behind generalization of fear and disproportionate intolerance of carnivores that rarely prey on livestock or people may minimize threats to such species. Because the striped hyena (hereafter hyena) is predominantly a scavenger, our a priori assumption was that hyena make a negligible contribution towards the total livestock depredation caused by its functional guild. We determined the socioeconomic status of villagers in an area of high HCC and examined whether the collective economic stress, and thus fear, caused by large carnivore predation on livestock leads to a generalized fear and intolerance of hyena. We also examined whether the loss of livestock due to disease plays a role in such generalizations.

## METHODS

### Study area

**FIGURE 1:**
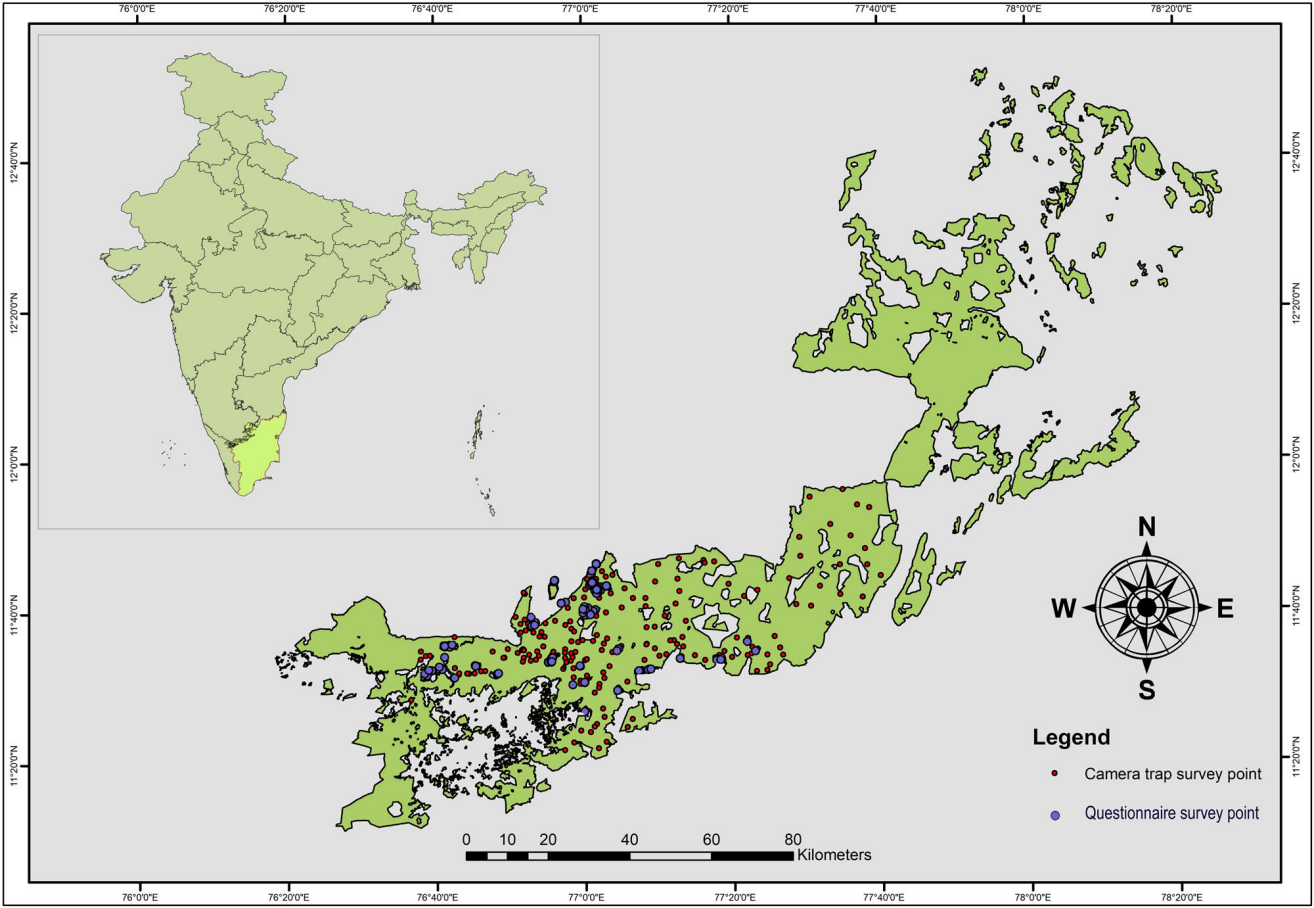
Location of 197 households surveyed on attitudes toward striped hyena conservation and locations of camera traps in the Western and Eastern Ghats parts of Tamil Nadu, India. Cameras were positioned to capture hyena and other co‐occurring species

The socioeconomic survey was conducted in the intensive study area (ISA) identified based on the current local distribution of hyena in the WEGPTN (Jhala et al., 2020). Initially, potential hyena distribution areas were marked over a large area based on a species distribution model calibrated with secondary data. This area was surveyed for animal sign and with camera traps (Figure 1). Camera traps were operated for 24 h for a minimum of 20 days in each location. Based on the recorded evidence of hyena occurrence, the ISA was delimited. The ISA encompassed most of the Sathyamangalam Tiger Reserve and Mudumalai Tiger Reserve. These reserves are in the Eastern and Western Ghats, respectively, and are connected landscapes that support the last population of hyena in its southernmost range in India. The area harbors a stable population of other predators, such as tiger, leopard, and dhole (Jhala et al., 2019; Venkatraman et al., 1995). Kurumbas, Sholagas, Irulas, and Ooralis are the major tribal communities inhabiting this area. They predominantly exhibit an agropastoralist lifestyle (Prabhakar, 2005).

### Socioeconomic survey

We administered semistructured questionnaire surveys in the Tamil language. We randomly selected agropastoralist households and interviewed the interested heads of households. For the survey, the heads of 197 households (Figure 1) were interviewed in 32 different settlements in and near the hyena's distribution range.

We assumed a priori that fear of penalty, expectation of reward, lack of understanding of questions, and false memory (Roediger & McDermott, 1995) of respondents would be the most probable causes of errors in our data. Therefore, we developed a quadruple check mechanism to maximize robustness of our data. First, to minimize their fear and reduce biased responses, we explained to respondents the purpose of the survey and clarified that it would have no immediate effects on management. Second, we interviewed only the heads of households who had lived in the study area for over 5 years, assuming that in 5 years they would be accustomed to carnivores and competent in ascertaining their kills. Third, we explained to each interviewee the signature kill pattern of all 4 carnivores that live in the area (e.g., laceration of flanks and hind legs for dhole and strangulation with throat bites for cats). Moreover, we showed them printed pictures of injury marks, scats, tracks, and rakes for all 4 carnivores. The pictures were developed for training field staff (Ramesh, 2010; Ramesh et al., 2012). Finally, to minimize the effect of false memory, we asked respondents to consider only predation that had occurred in the last 2 years.

The questionnaires had 3 broad sections (socioeconomic status, HCC, and attitude towards hyena). The socioeconomic attributes were (Table 1) annual family income from farming, wages, nontimber forest products, and dairy and cattle rearing. Cumulative family income was calculated by summing the amounts from these income sources. We asked for details on livestock lost in attacks by tigers, leopards, dholes, and hyenas and on livestock that died of disease in the last 2 years. Finally, respondents were asked whether they believed hyenas threaten their cattle and about their willingness to conserve the species in their vicinity. Survey questions are in Appendix S1.

**TABLE 1:** Variables used in calibrating random forest model to understand people's attitude toward striped hyena conservation

Covariate	Understanding	Variable type
Age	Age of respondent	Numeric
Gender	Gender of respondent	Binomial (male, female)
Community	Respondent is tribal or nontribal	Binomial (tribal, nontribal)
Literacy	A household member can read and write	Binomial (yes, no)
Annual income	Annual income of family	Numeric
Number of people employed	Total number of employed people in household	Numeric
Livestock dependency	Proportion of livelihood household earns from dairy and livestock rearing	Numeric
Forest dependency	Proportion of livelihood household earns by working with forest department	Numeric
Threat by hyena	Respondent perception of hyena as a threat to their livestock	Trinomial (yes, no, not sure)
Loss to disease	Estimated value of livestock mortality due to disease in last 2 years	Numerical
Loss to predation	Estimated value of livestock killed by carnivores in last 2 years	Numerical
Livestock vaccination	Family participation in government‐sponsored livestock vaccination scheme	Binomial (yes, no)
Total attack	Number of attacks on livestock by predators in last 2 years	Numeric

Research permission was obtained from the Office of the Chief Wildlife Warden, Tamil Nadu, under the provisions of the Wildlife Protection Act of 1972 and approved by Sálim Ali Centre for Ornithology and Natural History (SACON). Surveys were administered only to persons who provided verbal informed consent and agreed to proceed.

### Selection of variables

We selected 13 explanatory variables (Table 1), based on our understanding developed through relevant literature and open‐ended interaction with villagers, to test their effect on conservation attitude toward the species (Ballejo et al., 2020; Bhandari & Bhusal, 2017; Castillo‐Huitrón et al., 2020; Farhadinia et al., 2017; Gusset et al., 2009; Harihar et al., 2014). A Pearson correlation test was applied to all explanatory variables in R 3.0 (R Development Core Team, 2018), and among the highly correlated variables (*r* > 0.60) only the most ecologically sensible were retained. The total number of attacks on livestock and economic loss to predation were the only variables that were highly correlated, so the variable number of attacks on livestock was discarded (Appendix S2).

### Data analyses

Social systems are complex and include intricate and nonlinear interactions that result in uncertainty about phenomena associated with the system. Linear models are pragmatic, but they allow many assumptions that seldom stand true in a socioeconomic framework and that usually fail to recognize complex interactions. We used the random forest classifier (Breiman, 2001), which is a nonlinear model, to examine the importance of variables and their complex interactions in explaining the conservation attitude of villagers (Cutler et al., 2007). The binary response variable (conservation attitude) was class imbalanced (the number of cases of one class was substantially higher than another). Initially, more data were synthesized for the minority class with the majority weighted minority oversampling technique (Barua et al., 2012) in R. We built several random forest models in the random forest package (Liaw & Wiener, 2002) with a different number of trees to check prediction stability and grew 19,000 trees. To determine the optimal number of variables to be sampled randomly as candidates at each split (mtry), we ran several models with different values and found the model's highest accuracy at mtry = 4. We maintained this split in the final model. We kept tree depth (i.e., node size) at 1, which was the default value. We calculated 2 measures of variable importance: mean minimal depth, which is the depth of the node that splits on that variable and is the closest to the root of the tree, and Gini index (Lerman & Yitzhaki, 1984). The lower the value of mean minimal depth, the higher the importance of the variable in the model, and the higher the Gini index value, the greater the variable's importance. We also identified protagonist interactions of variables that explained the conservation attitude based on conditional mean minimal depth (Seifert et al., 2019). The accuracy of the calibrated model was checked by plotting the receiver operating characteristics (ROC) curve, which is a trade‐off between the proportion of correctly identified positive conservation attitudes (true‐positive rate) and wrongly identified negative conservation attitudes (false‐positive rate) in a data set. True‐positive and false‐positive rates were calculated on an out‐of‐bag data set (OOB). The OOB data refer to the data points from our data set that were not used in model calibration and were left out of model validation.

## RESULTS

**FIGURE 2:**
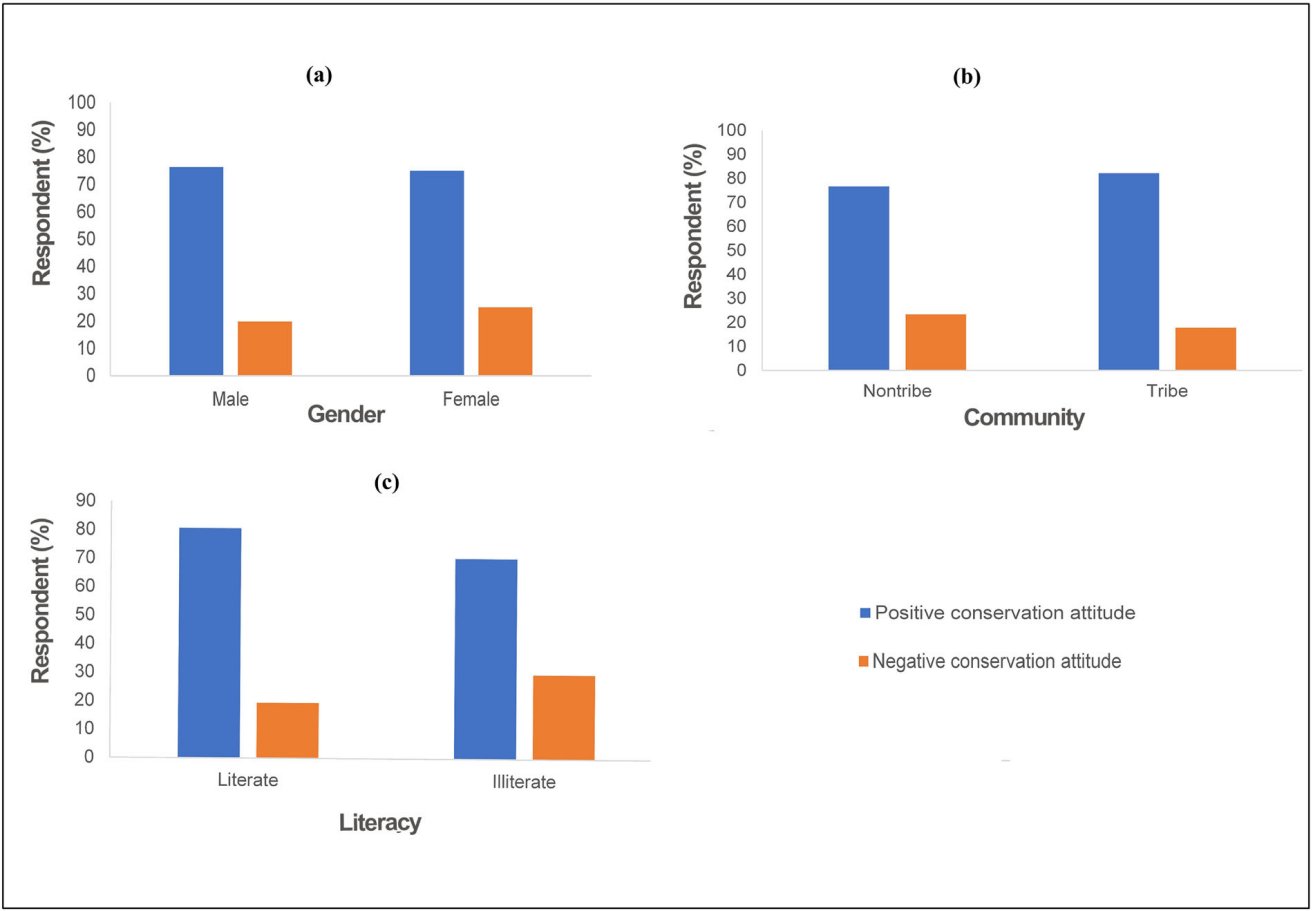
Attitude of (a) male and female survey respondents, (b) tribe and nontribe respondents, and (c) literate and illiterate survey respondents toward hyena conservation

We interviewed 45.69% tribal and 54.31% nontribal communities, and the proportion of male and female interview respondents was 82% and 18%, respectively (Appendices S3 & S4). Overall, 86% of households were literate. Literacy rates were 75% and 82% for tribal and nontribal people, respectively (Figure 2). People's livelihoods were heavily reliant on agropastoralism and wage‐based work. Annual mean dependency on animal husbandry as a livelihood was 27%. From 18% to 85% of people were dependent on forest‐department‐sponsored opportunities, but only 3% were beneficiaries of such opportunities. Overall, 69% (137 respondents) of respondents were uncertain about the predatory behavior of hyenas, 7% (14) perceived hyenas as a threat to their livestock, and 23% did not perceive hyena as a threat (Appendix S5). Analogous responses were observed across socioeconomic status of the respondent (Appendices S6–S8). Seventy‐nine percent of respondents were interested in conserving hyenas, whereas 21% were reluctant to conserve the species in their vicinity (Appendix S9). Furthermore, 23% of respondents who were uncertain about the predatory behavior of species were intolerant of hyena (Appendix S10). Nontribal respondents, women, and illiterate people showed higher relative intolerance for the species than their respective counterparts (Figure 2). Loss of cattle to disease caused 61% of respondents’ total economic loss, whereas predation caused only 40% loss. We recorded 245 instances of livestock predation reported by 40% of people interviewed. Out of these instances, hyenas were alleged to have been involved in 8 cases, and these included predation on 6 goats and 2 dogs.

**FIGURE 3:**
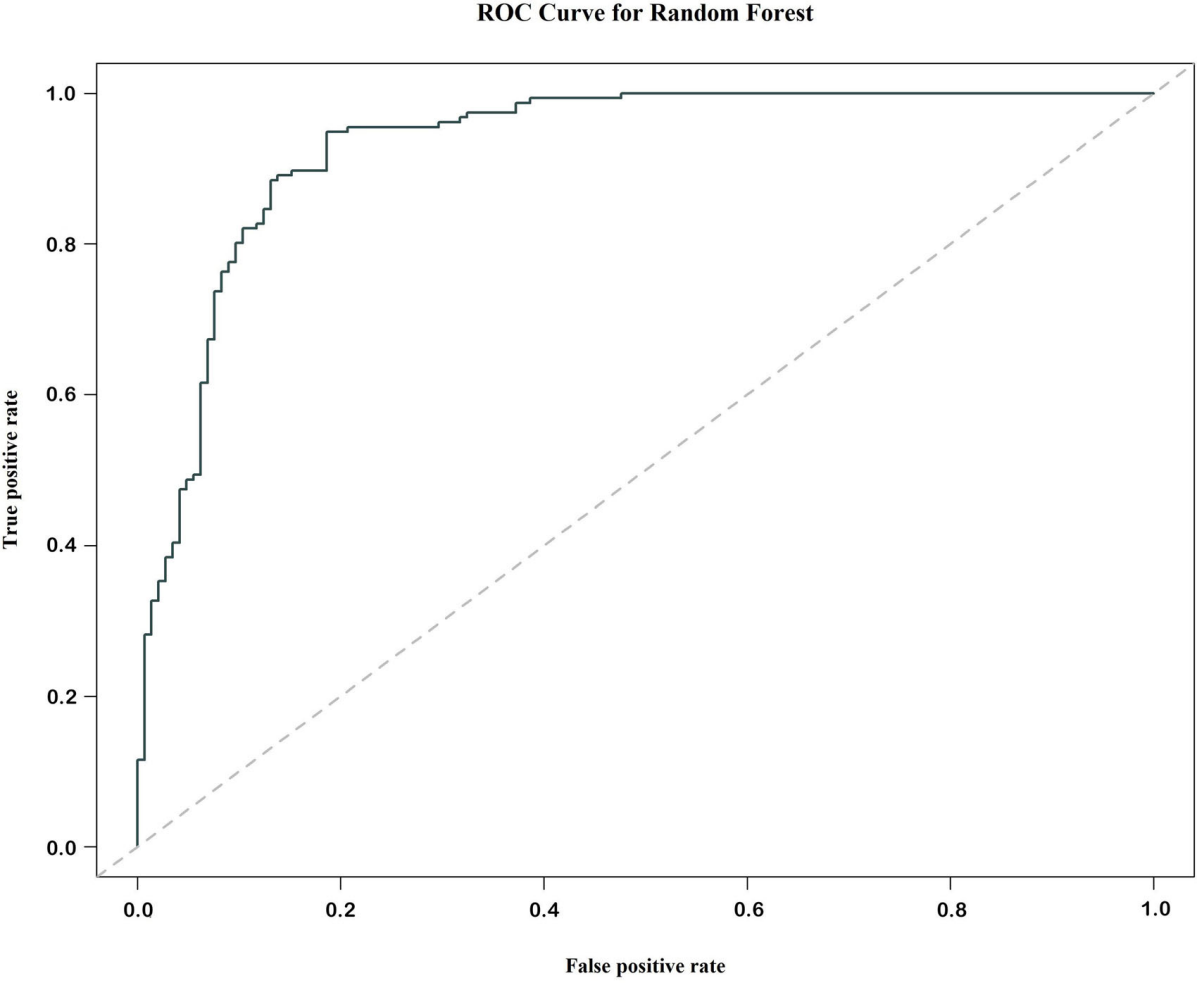
Accuracy of the calibrated model of people's attitude toward conservation of striped hyena, as indicated by the receiver operating characteristics (ROC) curve. Proportions are based on the number of predictive classifications made on out‐of‐bag data with different class thresholds based on the probability of positive conservation attitude predicted by the model (low value of false‐positive conservation attitude and high value of true‐positive conservation attitude correspond to a high likelihood of correct classification of test data run through the calibrated model)

**FIGURE 4:**
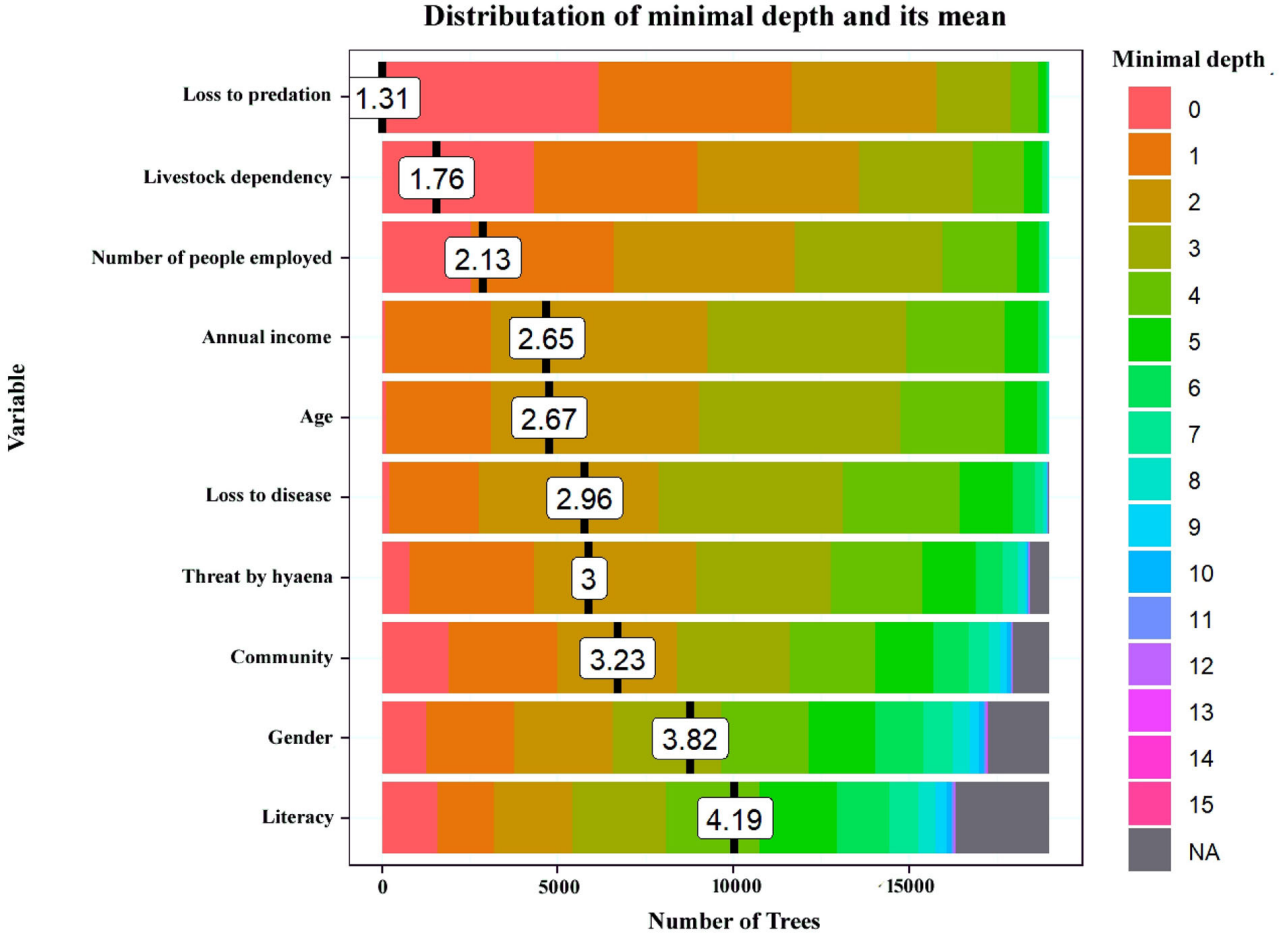
Variation in the importance of the 10 most important variables in determining survey respondents’ attitudes toward hyena, as indicated by mean minimal depth. The lower the value of minimal depth, the higher the robustness of the variable in making binary decisions. Mean minimal depth of a variable is the number of trees grown in the model (NA, no data because no trees were split on these variables for the corresponding ranges of numbers of trees)

The overall error rate of the calibrated model based on the OOB (data points not used in model calibration and left out for model validation) estimate was 12.62%. The false‐positive rate was 13.79% and false‐negative rate was 11.53% (Figure 3; Appendix S11). The calibrated model confirmed the loss to livestock predation as the most important in explaining the conservation attitude of respondents (Figure 4). The mean minimal depth and Gini index for the variable loss to predation were 1.31 and 30.87, respectively (Appendix S12). For both of these indices, livestock dependency was the second most important variable, followed by the number of people employed, annual income, age, loss to disease, threat from hyenas, community, gender, literacy, livestock vaccination, and forest department dependency (Figure 4; Appendix S12). Occurrence of no data (because no trees were split on these variables for the corresponding ranges of numbers of trees) in minimal depth of weak variables further suggested that weak variables appeared in fewer trees in comparison with strong variables (Figure 4). Based on the conditional mean minimal depth, 30 of the most influential variable interactions were identified (Appendix S13). Out of these interactions, the pair of variable loss to predation and livestock dependency were at the root of the tree. Other splitting nodes showed low conditional mean minimal depth. Thus, that interaction (i.e., variable loss to predation and livestock dependency) best predicted conservation attitude and affirmed the model results.

**FIGURE 5:**
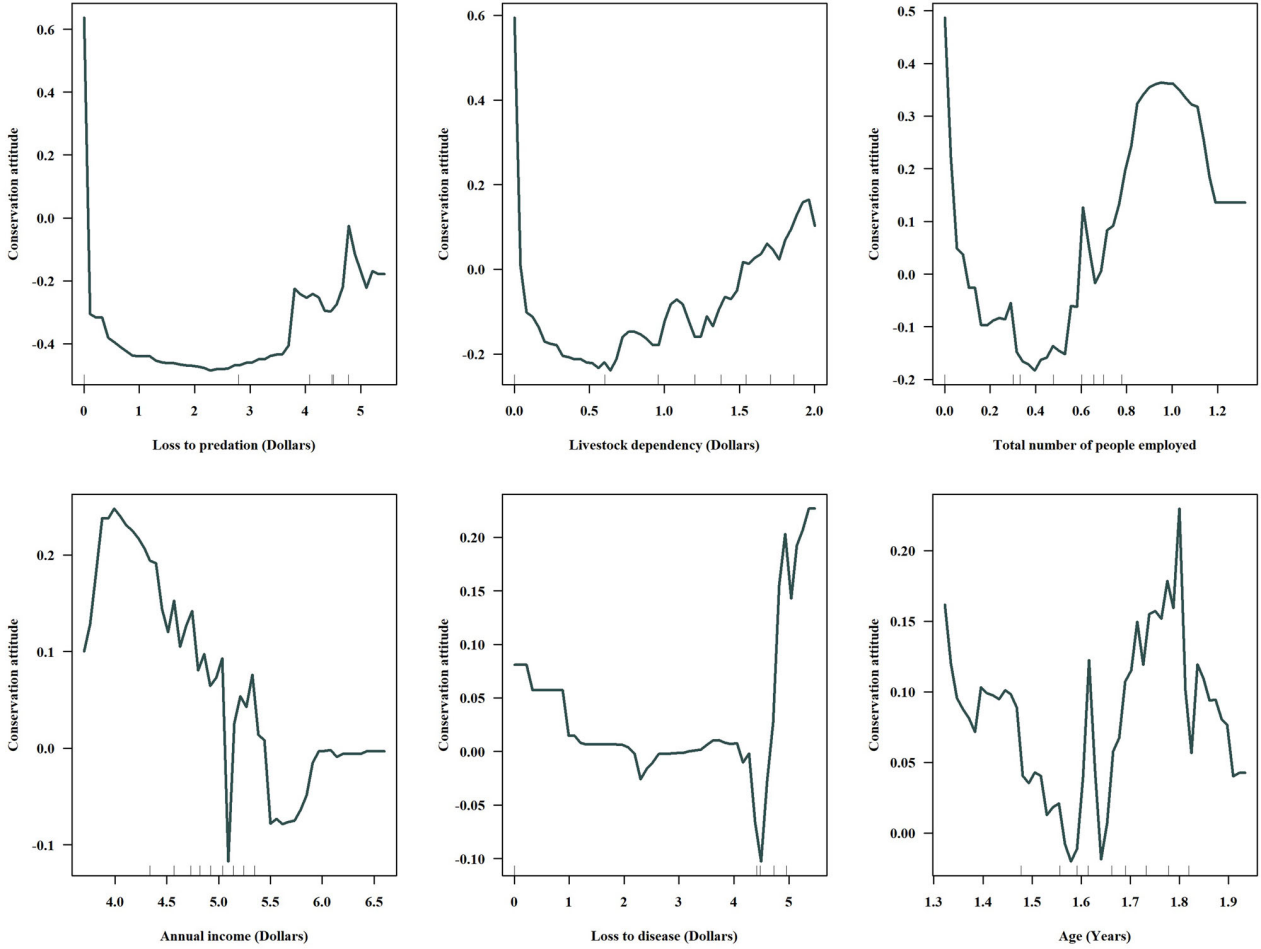
Marginal effect (i.e., how changes in the value of a variable when others are kept constant affect the likelihood for positive conservation attitude) of the 6 most important variables in a model used to predict conservation attitude of survey respondents

We plotted partial dependency graphs of the 6 most pivotal variables of the model (Figure 5). Loss to predation, livestock dependency, and annual income were negatively associated with conservation attitude. Thus, respondents were less likely to have a positive conservation attitude if the respondent was associated with a higher limit of these variables. Older respondents (>40 years) were more likely to possess a positive conservation attitude than their younger respondents. Similarly, the more people employed in a household, the more positive the conservation attitude. A negative conservation attitude was closely associated with perceptual uncertainty about the predatory behavior of hyena.

## DISCUSSION

Worldwide, mesocarnivores are persecuted based on their perceived threat to livestock, but these perceived threats rarely match the actual damage caused. Striped hyenas, which are persecuted (Bhandari & Bhusal, 2017; Selvaraj et al., 2018; Wagner, 2006), account for nearly negligible livestock loss relative to losses caused by sympatric species (Bopanna, 2013; Farhadinia et al., 2017; Srivathsa et al., 2019). Analogously, we found only a few cases of livestock predation by the species, and the majority of respondents (70%) were uncertain of the predatory efficiency of hyena. In our model, perceptual uncertainty was closely related to a negative conservation attitude. Such intolerance of uncertainty is not novel but a known tendency to perceive uncertainty as prejudicial (Carleton, 2016). In our study, perceptual uncertainty may be a result of the elusive and nocturnal behavior of hyena, which might reduce discriminative cognition and lead to the perception that 2 similar entities are the same. Leopards, which are similar to hyena in body size, have the highest livestock predation rate in this landscape (Ramesh et al., 2020). This morphological similarity probably means hyena were perceived as a threat, particularly by the people who were uncertain of the predatory behavior of hyena. In a similar case, irrespective of the damage inflicted, jackals (*Lupulella mesomelas*) were regarded as a threat by people who regarded leopards as problematic (Marker et al., 2003). Such stress‐induced generalization of fear is ubiquitous (Shin & Liberzon, 2010) and a contemporary theme in human psychology that regards such “perpetual enveloping” as a dispositional defensive tendency used to minimize threat exposure. Generalization was not limited to the perceived threat. About 21% of people were disproportionately intolerant of the species relative to the number of people who perceived hyena as a threat. Such a discrepancy was evident in a study that showed cheetahs (*Acinonyx jubatus*) are persecuted where people have a largely positive attitude toward the species (Marker et al., 2003). Indiscriminate removal of cheetahs was attributed to a traditional attitude, irrespective of contemporary conflict status of the species (Marker et al., 2003). The presence of secondary stimulus, play trees (i.e., trees used by cheetahs as a scent posts), in farmland that attracts other cheetahs could also affect the evaluation of primary stimuli and may be an alternative reason for the reported removal. Furthermore, in this study, collective economic stress from predation was a factor in determining intolerance and intolerance showed a positive relation with the cumulative loss inflicted by sympatric species. A similar pattern was observed by Farhadinia et al. (2017); a negative view toward leopards was strongly associated with the amount of loss caused by wolves. The observed generalization of intolerance associated with predation‐induced economic stress is a survival strategy and aligns with an increase in generalization as stress levels increase in both human and nonhuman animals (Dunsmoor et al., 2017). Generalization appears to be a defense mechanism that chooses “fear over tears” in seeking economic sustainabilityunder the high stress caused by livestock predation (Dunsmoor et al., 2017). We found that economic stability was undercut by livestock loses from disease more than by losses due to predation; thus, disease was also discerned as an important driver of generalization.

Acute intolerance of hyena conservation due to generalization of fear elicited under high economic stress was highly associated with livestock predation, livestock loss to disease, people's livelihood, and uncertainty regarding predatory efficiency. This generalization is situational and heavily conditioned on economic instability; so, there is leeway for its modulation with both fundamental and supplementary managerial interventions. Thus, to reduce intolerance, it is imperative to offset economic loss from livestock predation with prompt compensation. Veterinary support should be more available (Nawaz et al., 2016) in the study area and people should be acquainted with the scavenging behavior of the species. Compensation is expensive, particularly in low‐income countries where governance is poor (Nyhus et al., 2003), and can have counterproductive effects if management fails to provide compensation over a long term (Treves et al., 2009). Therefore, changing people's perceptions through community involvement (Treves et al., 2009) may help the management to mitigate the problem. In high‐income countries, community involvement has a firm place in conservation, apart from strong legislation (Woolaston et al., 2021). Such strategies have successfully lessened people's anger about depredation by wolves and helped people perceive conflict as a managerial lacuna in Washington (Anderson, 2021)

Efficient governance and strong legislation have also helped revive carnivore populations extirpated through persecution in the United States, Mexico, and Europe (Anderson, 2021; Castillo‐Huitrón et al., 2020; Chapron et al., 2014). Education and awareness are key to increase social acceptance of carnivores by changing the perception of the entire communities (Baruch‐Mordo et al., 2011). Therefore, we suggest that primary and secondary school students be provided with information about wildlife in the interface to reduce HCC. For this we suggest an apprise–appraise feedback framework to help in determining the effectiveness of awareness programs aimed at curtailing existing intolerance for hyena and establishing a firm place for perceptual uncertainty in management of HCC.

Pastoralists graze their cattle inside the forest, and this often results in livestock depredation by tiger, leopard, and dhole. Managerial interventions, such as stall feeding of cattle, would reduce predation and help assuage negative attitude towards carnivores. Reducing dependency on livestock by providing alternative opportunities, such as ecotourism and small‐scale handicrafts, in the area might also help in long‐term conservation of the last remaining major breeding population of hyena in southern India. There is a dire need for a demographic study of the population and identification of alternative habitats so that action can be taken to conserve this small and confined population.

We are probably the first to investigate fear‐induced generalization of predation risk to non‐obligate predators, although generalization is a common behavior in humans. We found an association between this type of generalization and economic instability and identified the importance of uncertainty in such misplaced generalization. Although fear‐induced generalization to the non‐obligate predator was prevalent, less than one fourth of respondents reported a negative conservation attitude towards the species. In our survey, 245 incidences of conflict were reported by 40% of respondents. This level of reporting could be a reason for the less reported negative conservation attitude, but intolerance among people may greatly increase if the rate of conflict increases. Even if 21% of the negative conservation attitudes were considered a nascent sign of threat generalization, the empirical model still soundly confirms the pattern. This implies that if the HCC continues to increase, it may severely imperil the existence of the last remaining major population of striped hyena in southern India.

We could not investigate species‐dependent variance in generalization due to the absence of other sympatric species in the study area. Again, in this study, we used the body size of hyena as an index of perceptual similarity to the obligate predator leopard, but it is not yet established empirically that how morphology and ecology of nonobligate predators determine species‐specific generalization (perceptual proximity). This conundrum can be examined relative to the theory of generalization, which can help to prioritize conservation based on the degree of perceived proximity of a species to obligate predators (generalization). With the addition of ecological and behavioral information on species, our methods can be used to check or predict if a wildlife species is prone to intolerance currently or in the future. Moreover, the quadruple check mechanism we used to minimize advertently and inadvertently induced differences in survey questions could have wide applicability to ensure fidelity of data in such studies.

## Supporting information

Supporting Information
Appendix Information
