# Supplementary material for: Generalization of threats attributed to large carnivores in areas of high human–wildlife conflict
Source: Conserv Biol. 2022 Aug 4;36(5):e13974. doi: 10.1111/cobi.13974 (PMC9805120; doi:10.1111/cobi.13974)

**Supporting Information**

**Appendix S1** Questionnaire for local community with respect to Human-carnivores conflict in Tamil Nadu, India

| Lat: Name of the interviewer:  Long: Date and Time:  Name of the nearest PA Distance from the nearest PA: | | | | | | |
| --- | --- | --- | --- | --- | --- | --- |
| **Section A. Personal details of the respondent** | | | | | | |
| 1.(a) Name | 1.(b) Age |  | 1.(c) Gender | M | F |  |
| 2. Name of the village: | | | | | | |
| 3. Contact number | | | | | | |
| 4. Ethnic group: Mountain Chetties/Kurumbas/Kattunaicker/Irula/ other minor group | | | | | | |
| 5. Annual income: | | | | | | |
| 6. Number of members can write and speak (Literate) | | | | | | |
| 7. Role in family | | | | | | |
| 8. Occupation of members   \| Occupation \| No. of person engaged \| Average annual income/person \| \| --- \| --- \| --- \| \| Working with Forest department \|  \|  \| \| Dairy products \|  \|  \| \| Wages/ Private \|  \|  \| \| Farming \|  \|  \| \| Collection and selling of NTFP \|  \|  \| \| Other (____________________) \|  \|  \| | | | | | | |
| 9. Access to Govt. schemes   \| Ration \| Medical facilities \| Human vaccination \| Loans \| \| --- \| --- \| --- \| --- \| \| Livestock vaccination \| Education \| Ujjwala Yojna \|  \| | | | | | | |
| **Section- B: Animal Husbandary** | | | | | | |
| 1.No. of livestock raised   \| Species \| Cow \| Buffalo \| Goat \| Sheep \| Pig \| Feral dogs \| Others \| \| --- \| --- \| --- \| --- \| --- \| --- \| --- \| --- \| \| Young \|  \|  \|  \|  \|  \|  \|  \| \| Sub-Adult \|  \|  \|  \|  \|  \|  \|  \| \| Adult \|  \|  \|  \|  \|  \|  \|  \| | | | | | | |
| 2(a). Where do they graze  2(b). Location | | | | | | |
| 3(a). Location of water hole for the livestock  3(b). How far is the water hole from your house | | | | | | |
| 4. Where does your livestock remain in the night hours and how is it protected | | | | | | |
| 5. List below the major threats to your livestock (Low, Medium, High)   \| Disease \| Predation \| Theft \| Others \| \| --- \| --- \| --- \| --- \| \|  \|  \|  \|  \| | | | | | | |
| 6. Whether any cases of diseases were observed in your livestock, If yes name the diseases   \| Sheep and goat pox(Chinam mai \| Foot and mouth  (kaal &Vaai punnu \| Leptospirosis  (Uruppu noi failure) \| Cow pox  (Madi noi) \| Vibrionic abortion  (nunu-uyeri-noi) \| \| --- \| --- \| --- \| --- \| --- \| \| Contagious bovine abortion  (kaukalaippu ) \| Mastitis  (Marbu noi) \| Black quarter  (karuppupulli noi) \| Anthrax \| Metabolic disease  (udal edai kudutha  koraiutha) \| \| Hemorrhagic septicaemia  (rattha sammanthapatta noi) \| Rabies \| Rinder pest  (komari noi) \| Others \| \| | | | | | | |
| 7.Are you aware of the insurance scheme YES NO | | | | | | |
| 8(a). Have you insured your cattle YES NO  8(b). List the reason for not insuring your cattle   \| Unaware \| Not interested to insure \| Don’t have money to afford \| \| --- \| --- \| --- \| \| Others \| \| \| | | | | | | |
| **Section C. Details of man-animal conflict** | | | | | | |
| 1.Your views on wildlife | | | | | | |
| 2. Is there presence of wildlife in your area; If yes name some of the mammals and how often you encounter them (weekly, biweekly, monthly, every six months, more than six months).   \| Species \| Presence \| Encounter rate \| \| --- \| --- \| --- \| \| Hyena \|  \|  \| \| Tiger \|  \|  \| \| Dhole \|  \|  \| \| Leopard \|  \|  \| \| Sloth bear \|  \|  \| \|  \| \| \| \| 3. Whether Hyena follows the herd YES NO \| \| \| \| 4. Whether Hyena goes for healthy/ sick one \| \| \| \| 5. Is there any wildlife conflict in your locality YES NO \| \| \| \| 6. Location of maximum conflict occurrence (*inside village, inside forest, adjacent to forest*) \| \| \| \| 7. Whether any carnivores found dead due to poisoning in recent years YES NO \| \| \| \| 8. If yes then specify the year and species \| \| \| \| 9.Do you think whether Hyena has reduced in last 5 years YES NO \| \| \| \| 10. Do you feel Hyena is a threat to you/ livelihood YES NO \| \| \| \| 11.Are you interested in conserving the species YES NO \| \| \| \| 12.Explain why \| \| \| | | | | | | |
| **Section E. Details of compensation** | | | | | | |
| 1. Are you aware of the compensation program YES NO | | | | | | |
| 2. Have you applied for the compensation, If yes fill the details below   \| Conflict animal \| Details of conflict \| Loss in rupees \| Amount compensated \| Average time taken for receiving compensation \| Whether the compensation provided is satisfactory/ not satisfactory \| \| --- \| --- \| --- \| --- \| --- \| --- \| \|  \|  \|  \|  \|  \|  \| \|  \|  \|  \|  \|  \|  \| \|  \|  \|  \|  \|  \|  \| | | | | | | |

| 3. Is the compensation scheme satisfactory, If no state the reasons why   \| Low amount paid as compensation \| Time delay in compensating \| Involves lots of traveling and spending money \| Attitude of forest department \| \| --- \| --- \| --- \| --- \| \| Longer procedure \| Others \| \| \| | | | | | |
| --- | --- | --- | --- | --- | --- | --- | --- | --- | --- | --- | --- | --- | --- |
| 4. Past scenario of conflict (Reduced, stayed same, increased)- If reduced or increased, state the reason | | | | | |
| 5.What do you suggest to be done to prevent conflict   \| Relocation of the animal \| \| Kill the conflict animal \| Physical barriers \| Noise/ Fire deterrent \| \| --- \| --- \| --- \| --- \| --- \| \| Electric fencing \| Others \| \| \| \| | | | | | |
| **Section F. General details of the respondent** | | | | | |
| 1. Mortality of livestock due to disease | | | | | |
| Year | 2018 | 2017 | 2016 | 2015 | 2014 |
| 1. No. cattle died due to ailments |  |  |  |  |  |

| 2. How are you dependent on the forest? | | | | |
| --- | --- | --- | --- | --- |
| a) NTFP | b) Water | c) Medicine | d) Religious need | e) Firewood |
| f) Herding livestock | g) Hunting | h) Fodder | i) Others |  |

| **Section G. Information on livestock predation by large carnivores** | | | | | | | | | |
| --- | --- | --- | --- | --- | --- | --- | --- | --- | --- |
| **Year & CattleNo.** | **Reason** | **Predator** | **Prey species**  **(B,C,D,G,S)** | **Age group of prey species** | **Time of conflict** | **MONTH** | **Location** | **Attack during grazing or stack-yarding** | **Attended/Unattended**  **(**UA, AA, CA, DA) |
| **2019** | Injured | Hyena |  |  |  |  |  |  |  |
|  |  | Leo |  |  |  |  |  |  |  |
|  |  | Dhole |  |  |  |  |  |  |  |
|  |  | Tiger |  |  |  |  |  |  |  |
|  | Killed | Hyena |  |  |  |  |  |  |  |
|  |  | Leo |  |  |  |  |  |  |  |
|  |  | Dhole |  |  |  |  |  |  |  |
|  |  | Tiger |  |  |  |  |  |  |  |
| **2018** | Injured | Hyena |  |  |  |  |  |  |  |
|  |  | Leo |  |  |  |  |  |  |  |
|  |  | Dhole |  |  |  |  |  |  |  |
|  |  | Tiger |  |  |  |  |  |  |  |
|  | Killed | Hyena |  |  |  |  |  |  |  |
|  |  | Leo |  |  |  |  |  |  |  |
|  |  | Dhole |  |  |  |  |  |  |  |
|  |  | Tiger |  |  |  |  |  |  |  |


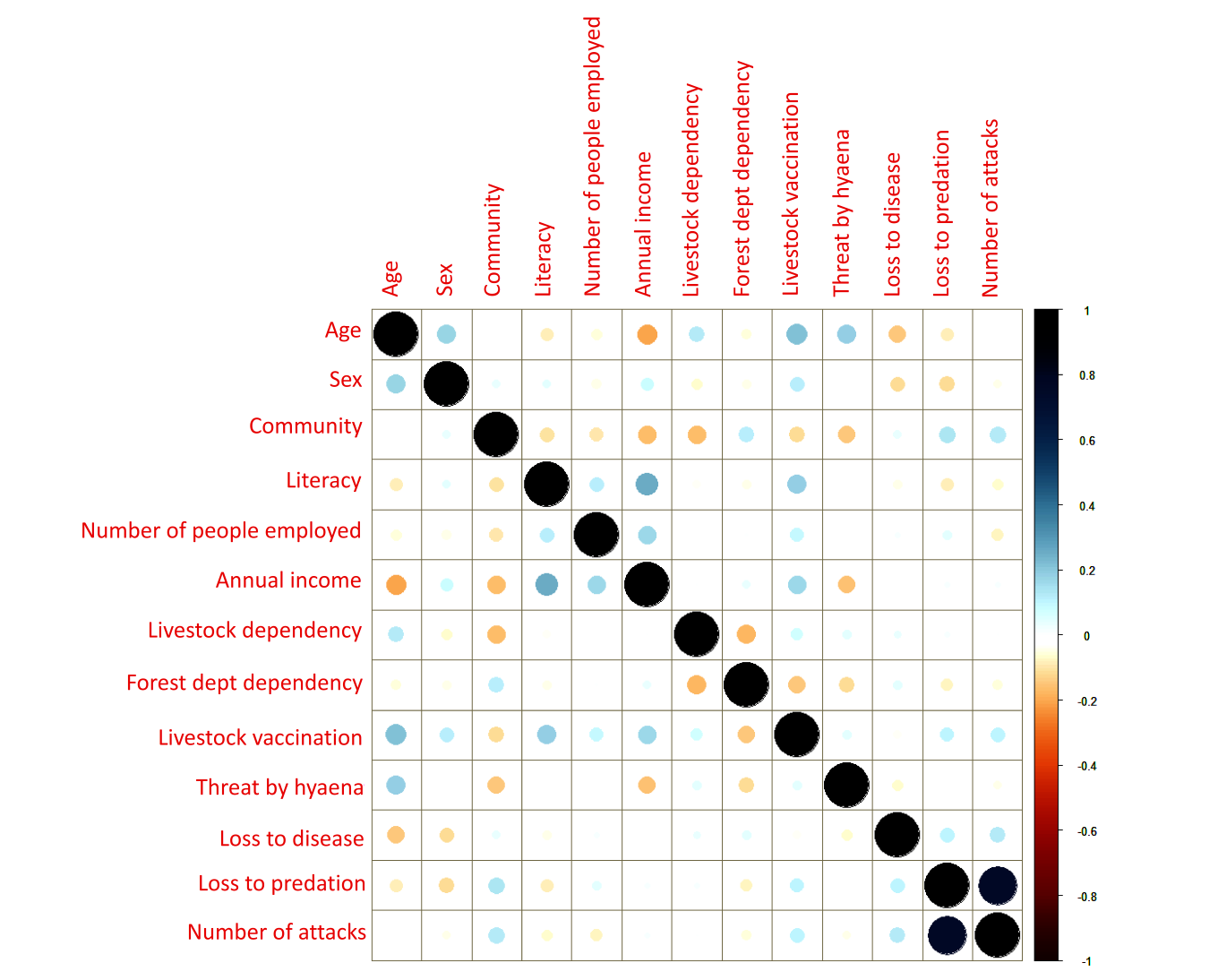
**Appendix S2** Correlation graph. Pearson correlation test was performed between 13 identified variables to check collinearity between the variables. Number of attacks by predators and economic loss to predation found to be highly correlated. Therefore, economic loss to predation was retained for the modelling and number of attacks was discarded.


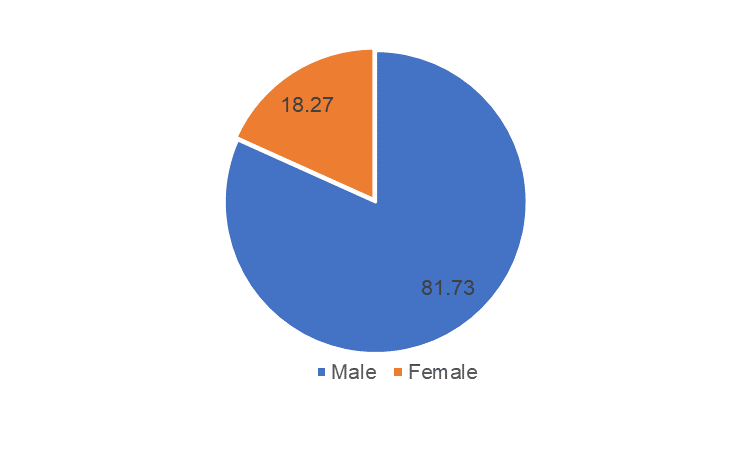
**Appendix S3** The percentage of male and female interviewed. During questionary survey, percentage of respondent was appeared to be male 81.73% (n=161) and female 18.27% (n= 36) respectively.


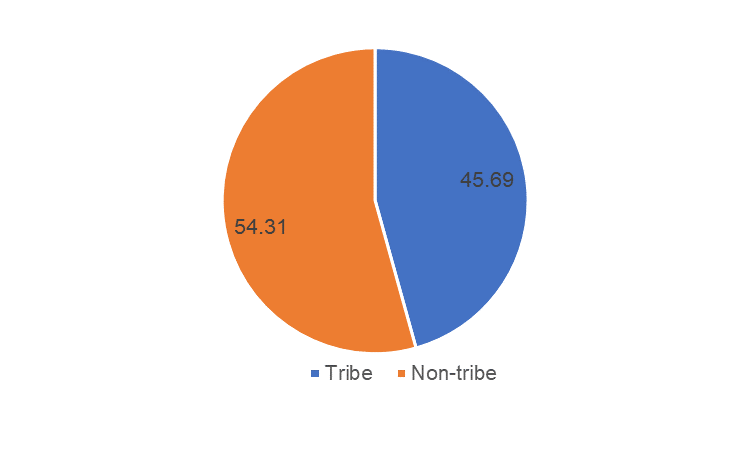
**Appendix S4** The percentage of tribe and non-tribe interviewed. During questionary survey, percentage of respondent was appeared to be non-tribe 54.31% (n=107) and tribe 45.69% (n= 90) respectively.

**Appendix S5** People's perception on predatory behaviour of hyaena in percentage. Multitude of respondent 69.54% (n=137) were ambiguous about predatory behaviour or efficiency of hyaena on livestock, 23.35% (n=46) perceived hyaena not as a threat and only 7.11% (n=14) considered hyaena as a threat for their livestock.


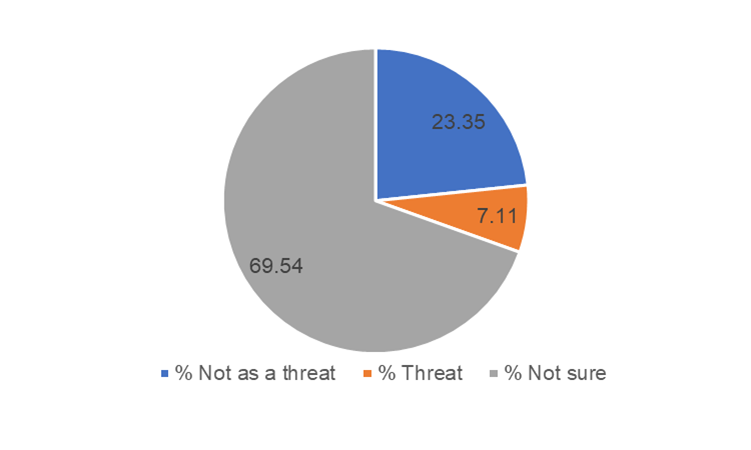


**Appendix S6** Frequency distribution graph of sex corresponding to predatory perception for hyaena. Most of people from both the classes (male 68.94% & female 72.22%) were not sure about predatory efficiency/behaviour of hyaena on livestock while only 8.07% of male and 2.78% of female reported hyaena as problematic or threat.

Correlation graph between the variable. Loss to predation and number of attacks by predators were found to highly correlated and number of attacks was from modeling.


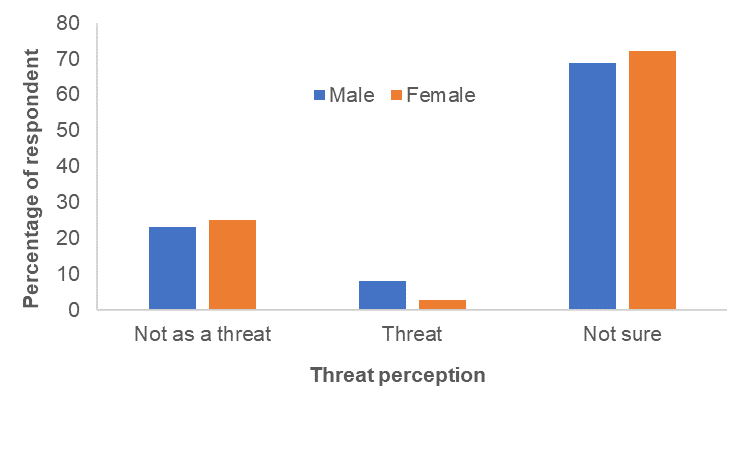


**Appendix S7** Frequency distribution graph of ethnicity (community) corresponding to predatory perception for hyaena. Most of people from both of the classes (tribal 62.22% & non-tribal 75.70%) were not sure about predatory efficiency/behaviour of hyaena on livestock while 8.89% of tribal and 5.61% of non-tribal respondent perceived hyaena as threat.


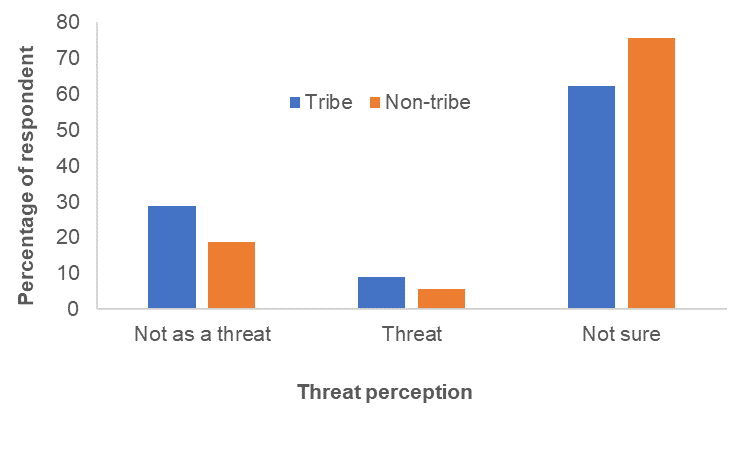


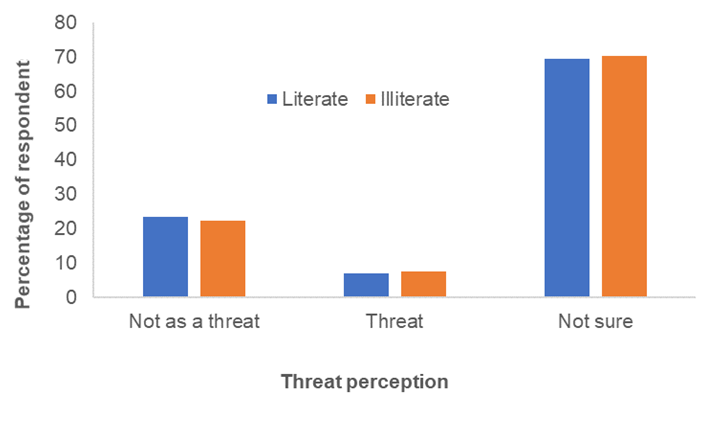
**Appendix S8** Frequency distribution graph of literacy corresponding to predatory perception for hyaena. Most of people from both the classes (literate 69.4% & illiterate 70.37%) were not sure about predatory efficiency/behaviour of hyaena on livestock while only 7.06% of literate and 7.41% of illiterate reported hyaena as problematic or threat.

**Appendix S9** Percent conservation attitude of people. During the questionary survey, 79.59% (n= 156) of respodent was found to be interested in conserving the species and 20.81% (n=41) were found to be not interested.


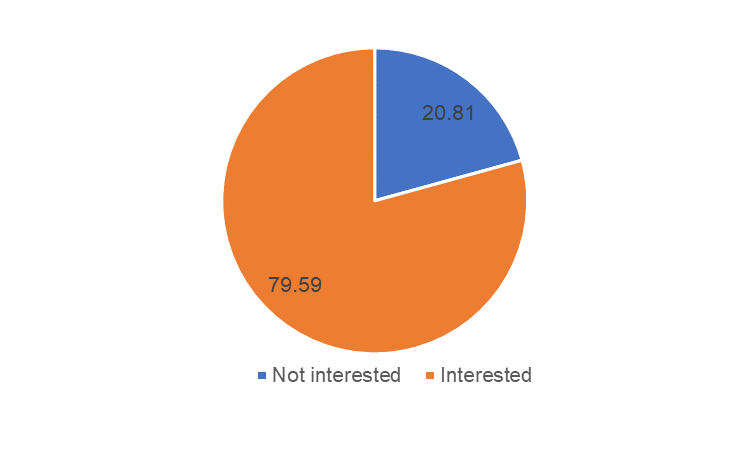


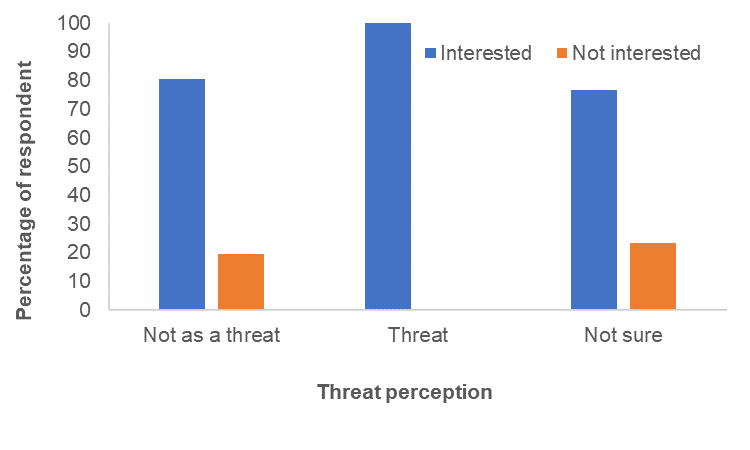
**Appendix S10** The graph representing percentage of interest for conservering the species corresponding to perceived threat for livestock. Across the perception, people who were uncertain about predatory behaviour of hyaena represented highest percentage of intolerance (23.36%) towards the species while all respondent who reported hyaena as a threat supported hyaena conservation.

**Appendix S11** Confusion matrix depicting the class error in the prediction made from random forest. Where model predicted 20 negative instances as positive and 18 positive instances were predicted as negative, therefore error for the negative was slightly higher as described in the main text.


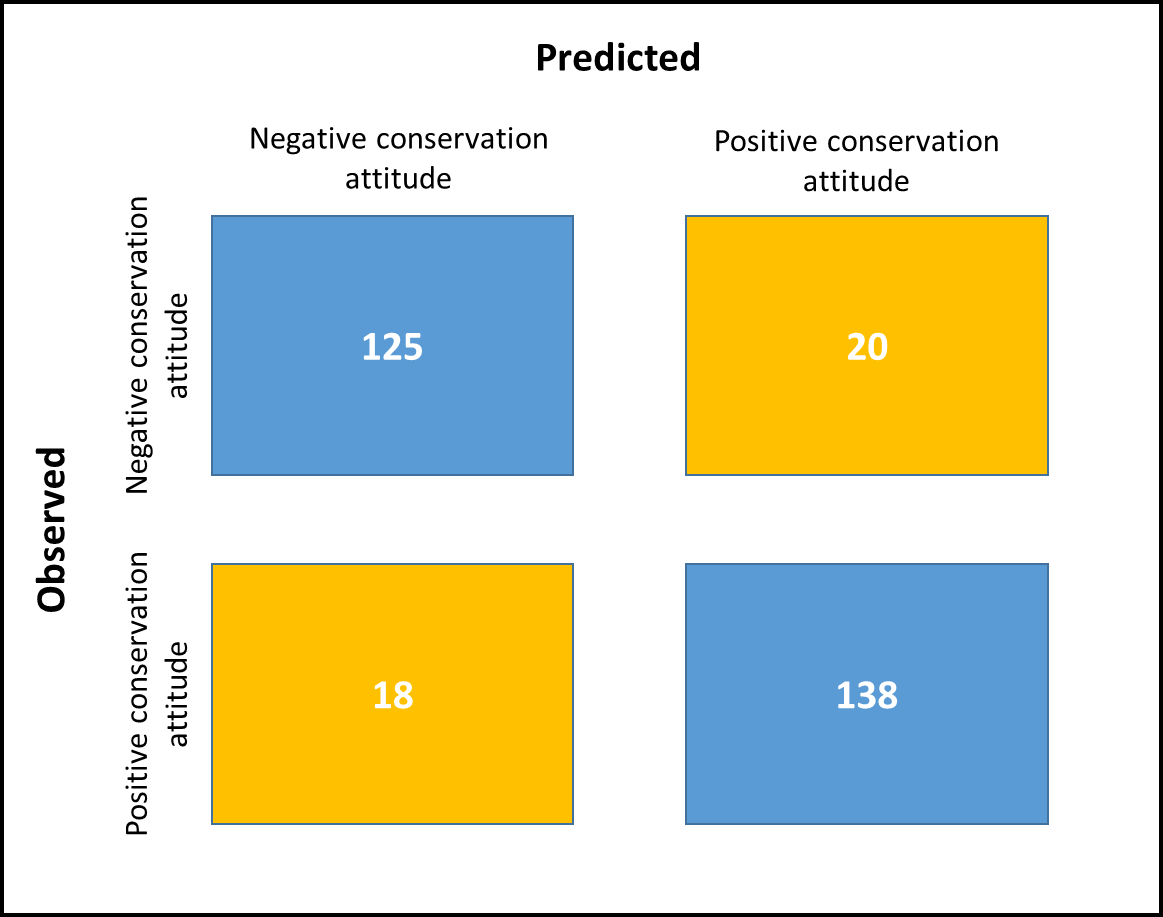


**Appendix S12** Values of two measures of variable importance Mean minimal depth and Gini index for the variables used for modelling. In the case of mean minimal depth, lower the value higher the importance in model but the case of Gini index higher the value higher the importance.

| **Variables used in the model** | **Minimal mean depth** | **Gini index** |
| --- | --- | --- |
| Age | 2.67 | 16.77 |
| Annual income | 2.65 | 16.81 |
| Community | 3.22 | 7.51 |
| Forest department dependency | 6.74 | 0.35 |
| Literacy | 4.18 | 4.79 |
| Livestock dependency | 1.75 | 23.73 |
| Livestock vaccination | 5.03 | 2.73 |
| Loss to disease | 2.96 | 12.98 |
| Loss to predation | 1.31 | 30.87 |
| Number of people employed | 2.13 | 18.65 |
| Gender | 3.82 | 5.13 |
| Threat by hyaena | 2.99 | 8.38 |

**Appendix S13** Best 30 interactions of variables according to mean of conditional minimal depth. Here occurrence correspond to the number of times the interaction appeared in the model.
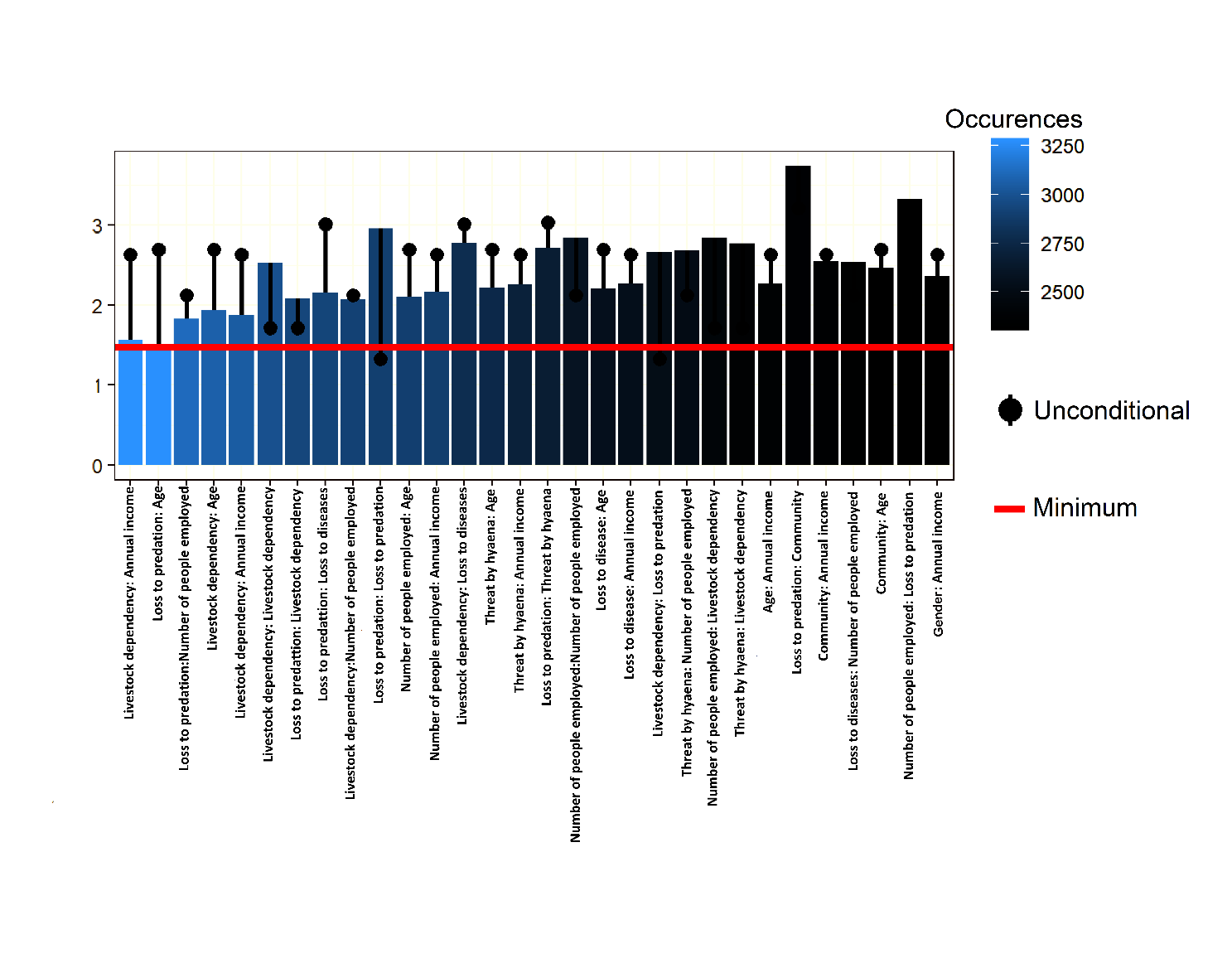

Supplement: Supplementary file 1 — Supporting Information Appendix Information [file COBI-36-0-s001.docx]
